# Different angular kyphosis locations have different relative positions of aorta to spine in patients with Pott’s deformity

**DOI:** 10.1186/s12891-022-05331-8

**Published:** 2022-04-20

**Authors:** Heng Jiang, Taotao Liao, Zhengyu Lu, Ce Wang, Rui Gao, Jun Ma, Xuhui Zhou, Jianquan Zhao

**Affiliations:** 1 Department of Orthopedics, Changzheng Hospital, Naval Medical University, Shanghai, People’s Republic of China; 2 Department of Orthopedics, Changzheng Hospital, Second Military University, No.415 Fengyang Road, Shanghai, People’s Republic of China

**Keywords:** Aorta, Pott’s deformity, Spinal tuberculosis, Kyphosis, Osteotomy

## Abstract

**Background:**

The position of the aorta relative to the spine in kyphosis secondary to Pott's deformity is little understood. The purpose of this study was to investigate the anatomic relationship between the aorta and the spine in patients with Pott’s deformity and to compare it with the normal people.

**Methods:**

Seventy-six patients with Pott’s deformity (Group TB) and seventy-two age- and sex-matched patients with a normal spine (group NC) were enrolled in this study. The relative position of aorta to the spine was evaluated from T4 to L4 on the computed tomographic angiography scans for controls and at the apex level for TB patient, and was classified into 4 kinds of degrees.

**Results:**

The left pedicle-aorta angle in group TB was significantly larger than that in group NC at the T6-L3 levels. Group TB exhibited significantly smaller left pedicle-aorta distance, pedicular line-aorta distance and vertebra/rib-aorta distance than those in group NC at the T5-T10 levels, but bigger at the L1-3 levels. Patients with grade 3 and 4 aorta had more segments involved compared with those with grade 1 aorta. Patients with grade 2, 3, and 4 aorta showed larger kyphotic angles than those with grade 1.

**Conclusions:**

Patients whose morbid segments involved only thoracic vertebrae presented with an “Ω” shaped aorta in sagittal plane, and 4 different kinds of degrees of aorta relative to the vertebra/rib in axial plane. Patients whose morbid segments covered lumbar vertebrae presented with an “M” shaped aorta in sagittal plane, and the aorta shifted further from apex vertebra but was located in close proximity to the vertebral body at levels above and below the osteotomy levels in axial plane.

## Background

Spinal tuberculosis is the commonest manifestation of extra-pulmonary tuberculosis, accounting for nearly half of patients with skeletal tuberculosis [1]. The chronic infection leads to the anterior structural destruction and collapse of the vertebral body, which eventually gives rise to various degrees of sagittal plane deformity, angular kyphosis (Pott’s deformity) greater than 100 degrees in extreme cases. Severe kyphotic deformity may result in back pain, neurologic deficits, pulmonary function deficiency, self-image and psychological problems, which require surgical intervention.

For the healed stage of spinal tuberculosis, Pott’s deformity can only be corrected by three-column osteotomies and several osteotomy techniques have been used [2]. These include the pedicle subtraction osteotomy (PSO) [3], a closing wedge osteotomy; closing-opening posterior vertebral column resection (PVCR) [4]; vertebral column decancellation (VCD) [5]; and transpedicular bi-vertebrae osteotomy technique [6]. Most of these techniques need three-column exposure of the spine and involve resection of one or multiple vertebral segments and ribs (when operated in thoracic segments) [7]. The aorta is vulnerable to be injured during the exposure and resection of the bony components, especially when the post-infectious fusion mass tether the aorta to the anterior structures of the spinal column or the rib [1, 8].

Some previous studies have evaluated the position of the aorta relative to the spine in kyphosis secondary to ankylosing spondylitis [9]. We assumed that the destruction and fusion of the vertebral bodies in Pott's deformity may contribute to the variation of the position of the aorta to spine. Therefore, the objective of this study was to investigate this anatomic relationship and to compare it with the normal people.

## Methods

**Fig. 1:**
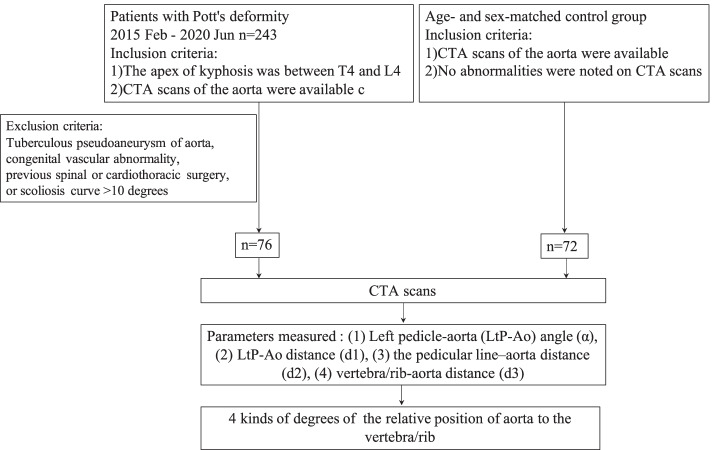
Flowchart of included subjects for analysis

The medical records of 243 patients with Pott’s deformity in our center undergoing correction surgery between February 2015 and June 2020 were retrospectively reviewed. Diagnosis was made based on laboratory tests, radiographic imaging and histopathology. The inclusion criteria were as follows: (1) the apex of kyphosis was between T4 and L4; (2) computed tomographic angiography (CTA) scans of the aorta were available. Patients with tuberculous pseudoaneurysm of aorta, congenital vascular abnormality, previous spinal or cardiothoracic surgery, or scoliosis curve > 10 degrees were excluded. Meanwhile, 72 age- and sex-matched controls were included into this study, who underwent CTA scans because of non-spinal pathologies. Patients were excluded if any abnormalities, such as spine-related diseases and vascular malformation, were noted on CTA scans (Fig. 1). This study was approved by the ethical committee of Shanghai Changzheng hospital and written informed consent was obtained from all individual participants included in the study.

### Radiographical measurements

For patients with Pott’s deformity, the standing anteroposterior and lateral radiographs were performed preoperatively, and kyphotic angle, sagittal vertical axis were determined. The apex of the kyphotic curve and the morbid segments of each patient were recorded. Disability status was assessed using the Oswestry Disability Index (ODI).

### Computed tomographic angiography measurements

To quantify the relative position of the aorta and spine, a Cartesian coordinate system was built up on the axial CT scans of the vertebral bodies according to the methods of Takeshita et al [10]. And the following parameters were measured at each level from T4 to L5 in group NC and apex level in group TB: (1) Left pedicle-aorta (LtP-Ao) angle (α), subtended by the y-axis and a line connecting the origin and the center of the aorta. (2) LtP-Ao distance (d1), defined as the distance between the origin and the edge of the aortic wall. (3) the pedicular line–aorta distance (d2), defined as the distance from the edge of the aorta to the x-axis. (4) vertebra/rib-aorta distance (d3), defined as the minimum distance from the edge of the aorta to vertebra or rib. These parameters were measured by 2 observers blinded to the information of the subjects.

**Fig. 2:**
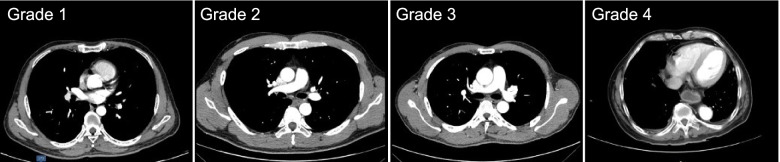
The average left pedicle-aorta angle, left pedicle-aorta distance, the pedicular line-aorta distance and the vertebra/rib-aorta distance from T4-L3 levels for patients in group TB (dark bars) and patients in group NC (T4-L4, light bars)

From the axial images of CTA scans, we selected the vertebral segments with the minimum value of the pedicular line-aorta distance in group NC and classified them into three kinds of degrees based on the relative positions of aorta to the vertebra/rib. In grade 1, the aorta was seen at the antero-middle position of the vertebral body or antero-lateral position of the first third of the vertebral body; in grade 2, the aorta moved posteriorly from the first third of the vertebral body to the costal head; in grade 3, the aorta was located laterally to the costal head, the costal neck and the costal tubercle (Fig. 2). We evaluated the CTA images of patients with Pott’s deformity and found that in some patients, the aorta moved positively and laterally and was located in vicinity of the rib (Fig. 2), which we defined as grade 4.

### Statistical analysis

The data were analyzed using SPSS version 13.0 for Windows (SPSS Inc., Chicago, IL). The interrater reliability of two observers’ measurements was assessed using intraclass correlation coefficient, which was calculated using 2-way mixed intraclass correlation coefficient models (3, k). The data were tested for normal distribution with the Shapiro–Wilk test, and an independent sample t test was performed to determine the differences between the 2 groups. And Kruskal–Wallis test was used to determine the differences between the 4 subgroups. Significance was defined as a *P* value of less than 0.05.

## Results

A total of 76 patients with Pott’s deformity (group TB) and 72 normal subjects (group NS) who satisfied the aforementioned criteria were enrolled in this study. The group TB was composed of 29 females and 47 males with an average age of 48.8 years (range, 26–64 yr), and the group NC consisted of 27 females and 45 males with a mean age of 47.3 years (range, 25–76 yr). With respect to age or sex distribution, there was no significant difference between the 2 groups. The average coronal Cobb angle was 5.6° (range, 0–9°), and the average thoracic, thoracolumbar, and lumbar kyphosis was 113.8° (range, 73.1–154.8°), 112.0° (range, 77.1–145.6°) and 98.0° (range, 52.7–155.0°), respectively. Interrater reliability was found to be high for each measure (Table 1).

**Table 1:** Quantitative measures and interrater reliability

Parameters	Interrater Reliability^a^
Left pedicle-aorta (LtP-Ao) angle (α)	0.87 (0.82 to 0.90)
LtP-Ao distance (d1)	0.92 (0.85 to 0.95)
The pedicular line–aorta distance (d2)	0.93 (0.88 to 0.96)
Vertebra/rib-aorta distance (d3)	0.90 (0.84 to 0.92)

^a^The values are given as the intraclass correlation coefficient, with the 95% confidence intervals (CI) in parentheses

**Fig. 3:**
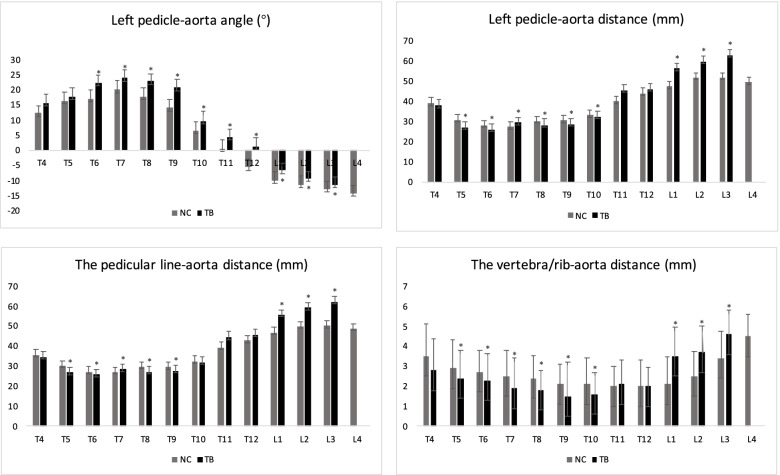
The average course of the aorta relative to the spine in the group TB (red points) and group NC (blue points) in Cartesian coordinate system. Compared with the normal subjects, the aorta in patients with Pott’s deformity migrated to a posterior-lateral direction relative to the vertebrae at the T5–10 levels. At the L1-3 levels, the aorta was shifted further from vertebral body

**Fig. 4:**
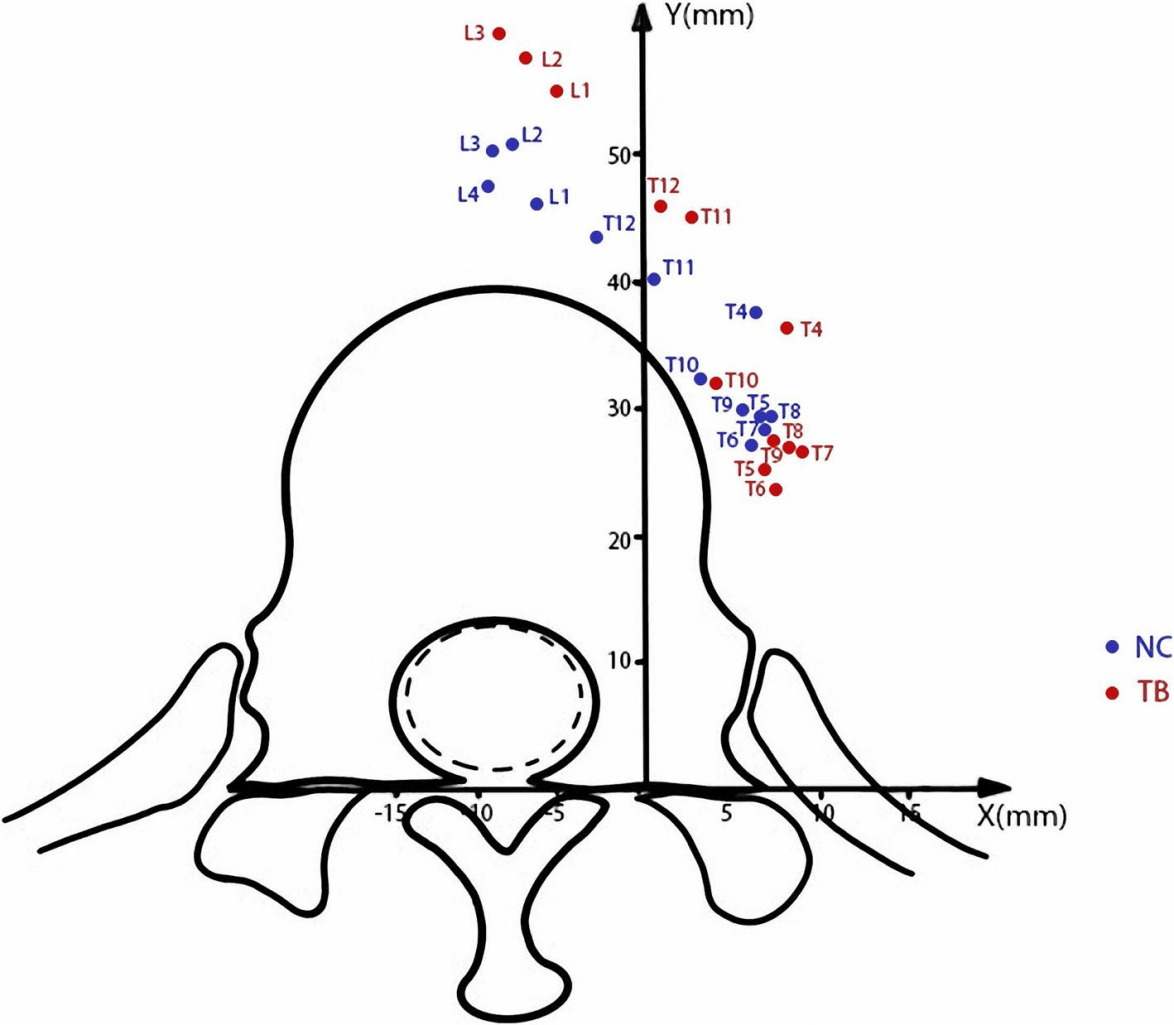
Different kinds of courses or shapes of aorta in patients with different kyphotic segments

**Fig. 5:**
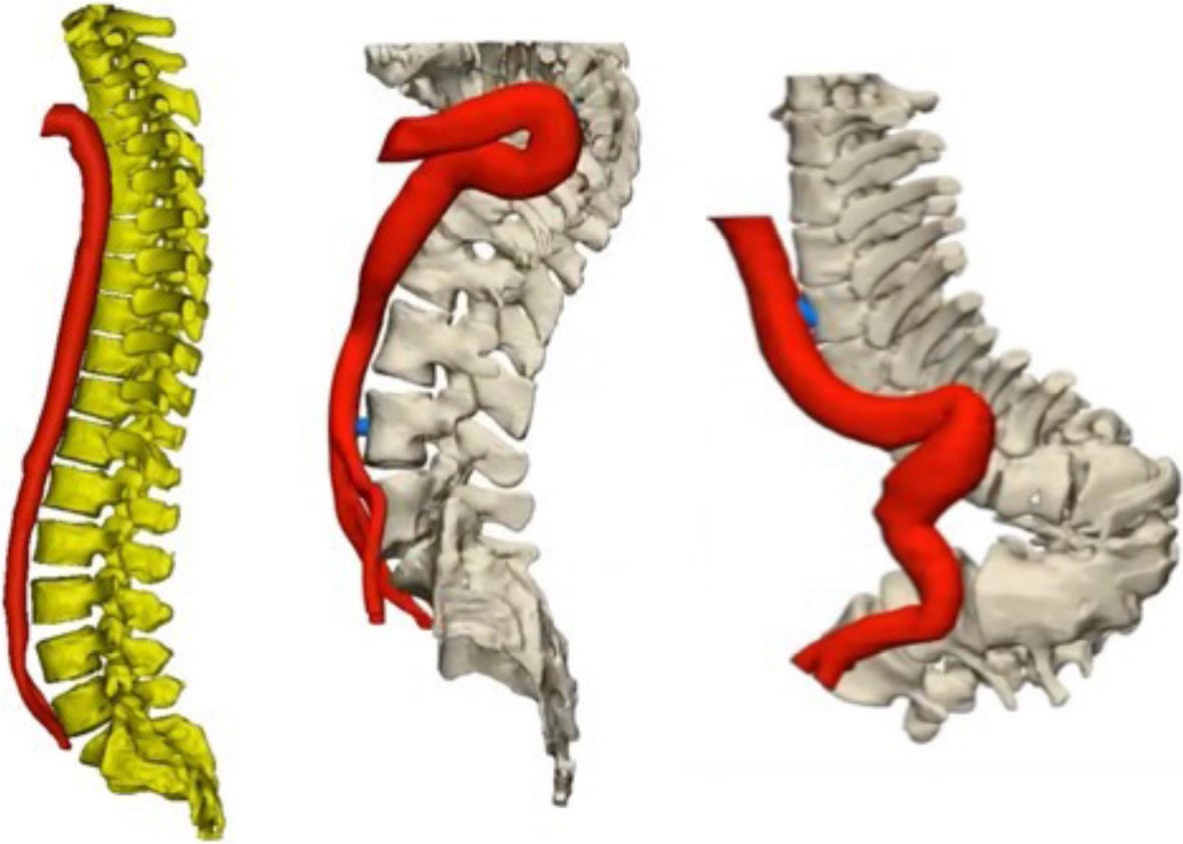
Different relative positions of aorta to the vertebra/rib in axial CT plane

In group TB, there were 10 patients with apex vertebra located at T7, 11 patients at T11 and 9 patients at L3. Generally, the α angle in group TB was significantly larger than that in group NC at the T6-L3 levels (*p* < 0.05) (Figs. 3, 4). Group TB exhibited significantly smaller d1, d2 and d3 than that in group NC at the T5-T10 levels (*p* < 0.05, Figs. 3, 4), While they showed longer d1, d2 and d3 than that in group NC at the L1-3 levels (*p* < 0.05, Figs. 3, 4 and 5).

**Fig. 6:**
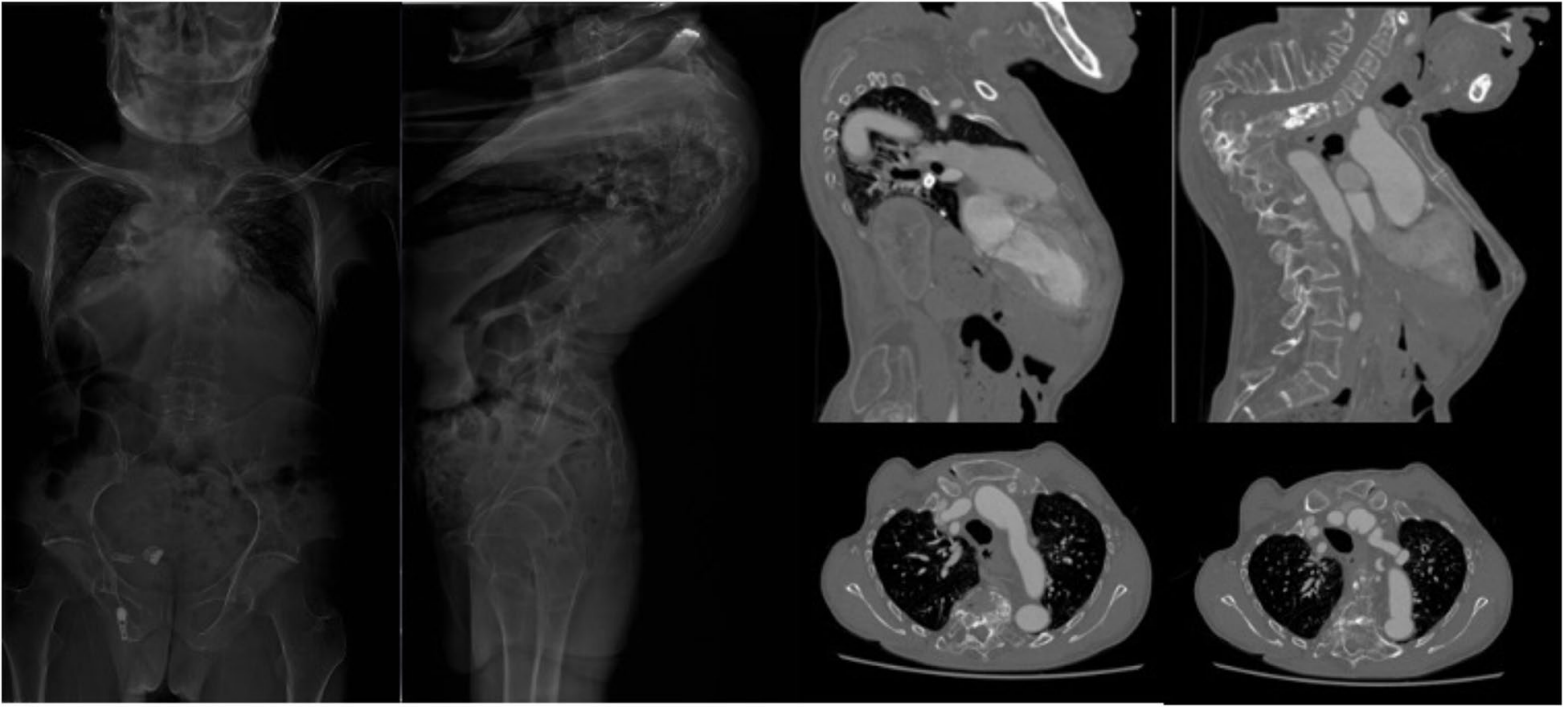
A 55 years old patient presented with lower limb weakness. Anterior column collapse in upper thoracic region. The kyphotic angle of T4-6 was 73.1° and SVA was measured 1.7 cm. CT scans showed that the aorta was located at the antero-lateral position of the first third of the apex vertebra (Grade 1)

**Fig. 7:**
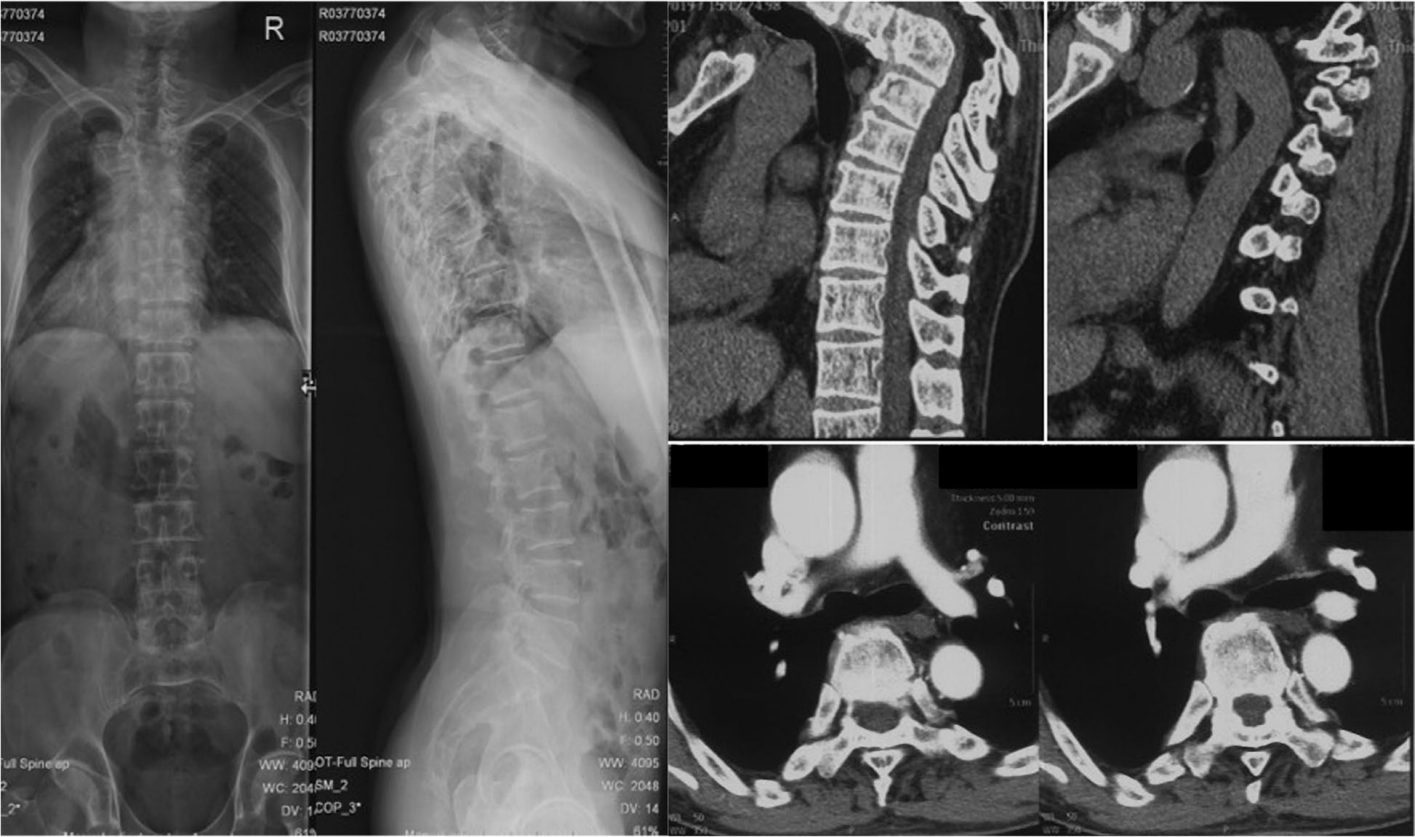
A 53 years old patient presented with back pain and shortness of breath. The kyphotic angle of T3-11 was 79.2° and SVA was measured 2.2 cm. CT scans showed that the aorta was seen near the lateral-wall of the apex vertebra and in front of the costal head (Grade 2)

**Fig. 8:**
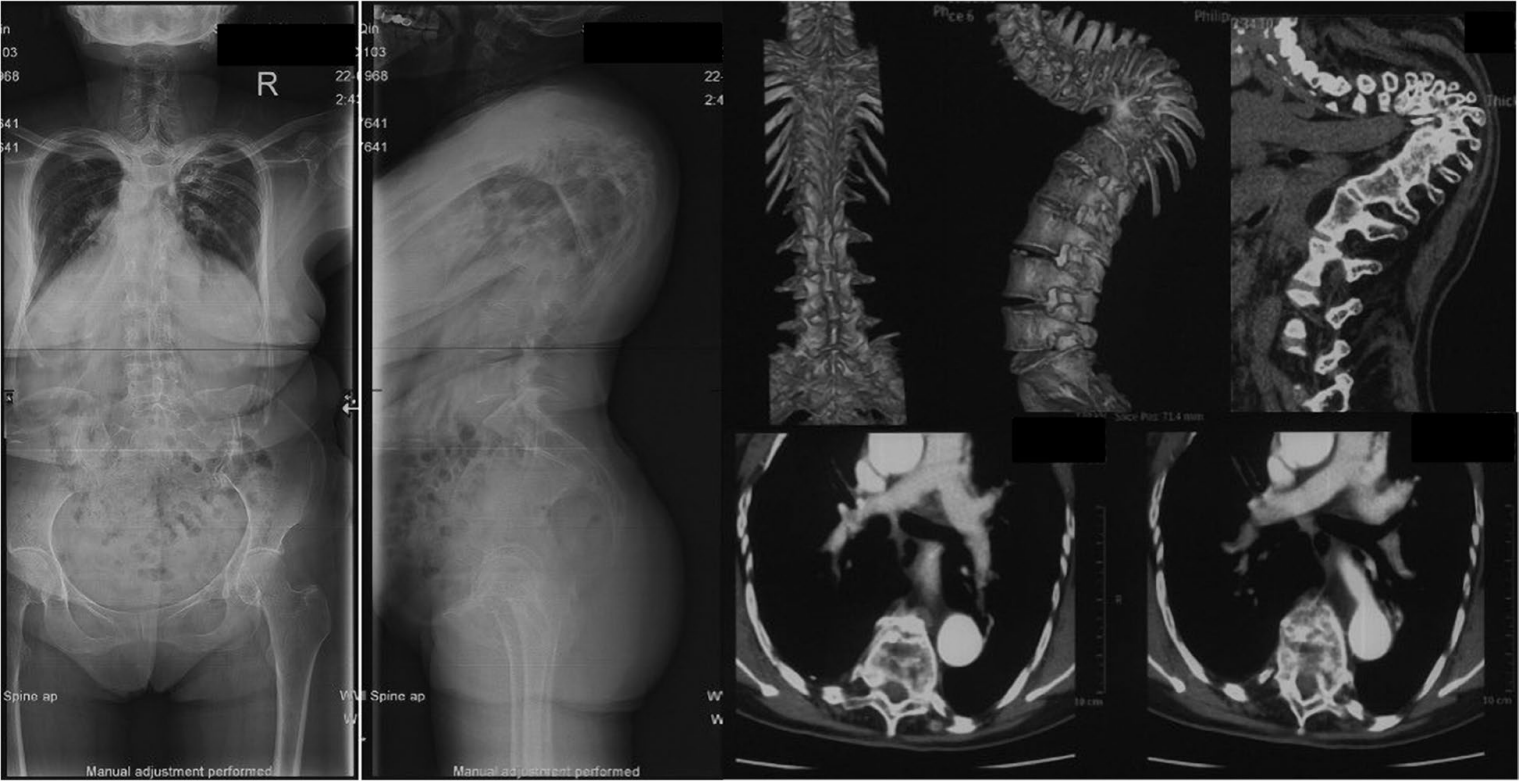
A 59 years old patient presented with weakness and numbness of left lower limb. The kyphotic angle of T2-6 was 111.1° and SVA was measured -2.2 cm. CT scans showed that the aorta was located laterally to the costal head and the costal neck at apex vertebral level (Grade 3)

**Fig. 9:**
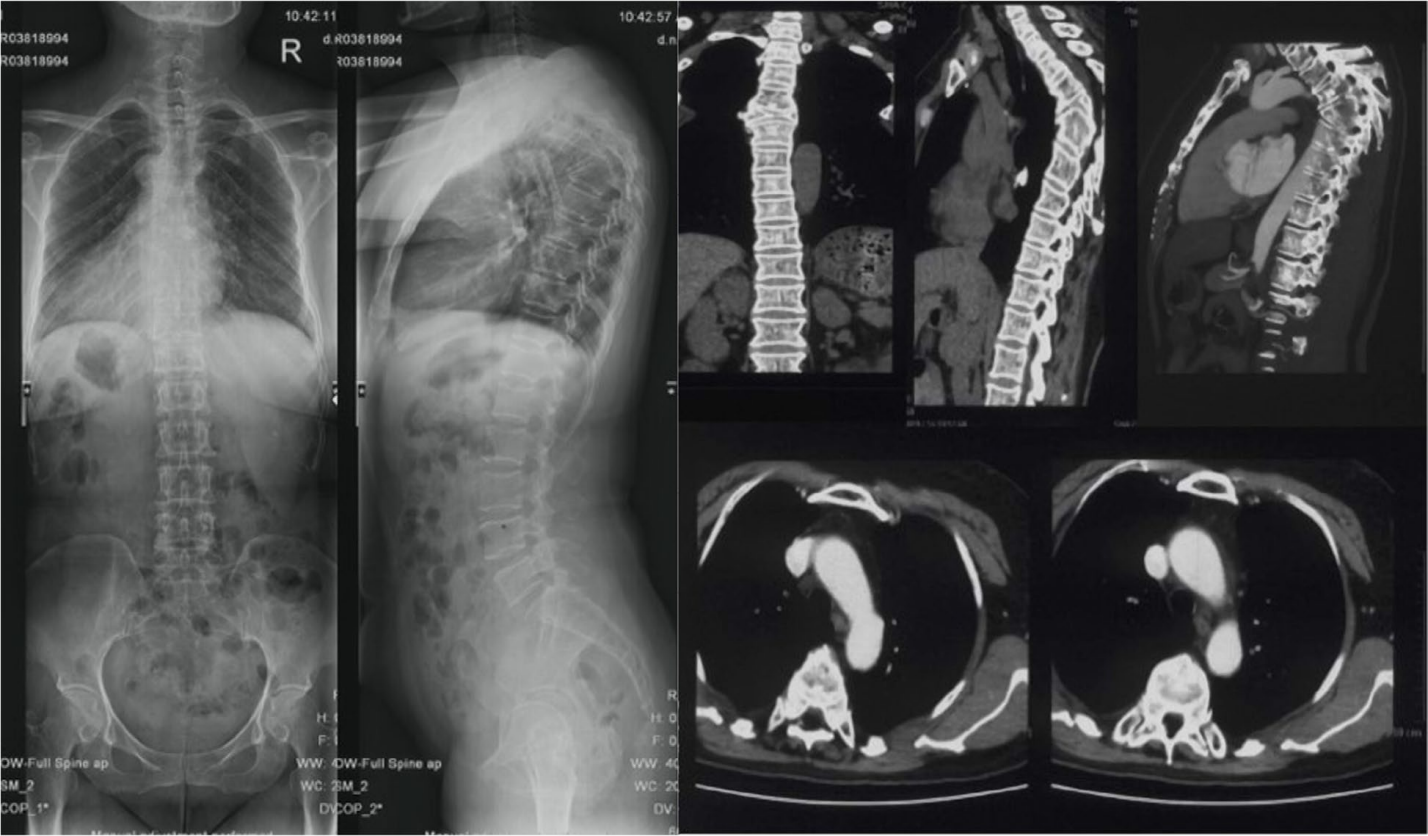
A 64 years old patient presented with weakness and pain of lower limbs. The kyphotic angle of T5-11 was 120° and SVA was measured -1.0 cm. CT scans showed that the aorta was located in vicinity of the rib at apex vertebral level (Grade 4)

There were 32 (44.4%) subjects in group NC with grade 1 aorta, 30 (41.7%%) with grade 2 aorta and 10 (13.9%) with grade 3 aorta. There was no significant difference between the 3 subgroups in terms of ages, gender, vertebral segment with minimum value of d2 or d3 (Table 2). In patients whose morbid segments of Pott’s deformities did not involve the lumbar region, there were 7 (15.5%) with grade 4 aorta (Fig. 6), 13 (28.9%) with grade 3 aorta (Fig. 7), 17 (37.8%) with grade 2 aorta (Fig. 8) and 8 (17.8%) patients with grade 1 aorta (Fig. 9). There was no significant difference between the 4 subgroups in terms of gender, age of kyphosis onset, disease duration, apex level, d3, SVA or ODI score. Patients with grade 3 aorta and with grade 4 aorta had more segments involved compared with those with grade 1 aorta (*p* = 0.0006 and *p* < 0.001, respectively). Patients with grade 2, 3, and 4 aorta showed larger kyphotic angles than those with grade 1 (*p* = 0.0013, *p* = 0.0008 and *p* = 0.0002, respectively) (Table 3).

**Table 2:** Relative positions of aorta to the vertebrate and the rib in control subjects

	Grade1 *n* = 32	Grade2 *n* = 30	Grade3 *n* = 10
Age (yrs)	46.1 ± 9.7	47.8 ± 8.6	46.6 ± 10.3
Gender			
Male	19	20	7
Female	13	10	3
Vertebral segment with minimum value of the pedicular line-aorta distance	6.6 ± 0.8	6.9 ± 0.5	6.5 ± 0.8
The vertebra/rib-aorta distance (mm)	1.5 ± 0.8	1.6 ± 1.1	1.8 ± 0.9

**Table 3:** Relative positions of aorta to the apex of the kyphotic curve in group TB

	Grade1 *n* = 8	Grade2 *n* = 17	Grade3 *n* = 13	Grade4 *n* = 7
Age of kyphosis onset (yrs)	14.4 ± 9.5	14.4 ± 8.2	14.2 ± 5.6	14.3 ± 7.0
Disease duration (yrs)	37.1 ± 10.7	37.9 ± 12.5	38.8 ± 7.6	39.0 ± 22.0
Gender				
Male	6	13	8	5
Female	2	5	5	2
Segments involved (n)	2.8 ± 0.5	4.3 ± 1.6	5.1 ± 0.6^b^	6.3 ± 0.6^c^
Apex level	7.8 ± 2.7	7.9 ± 1.8	6.7 ± 2.7	7.8 ± 1.7
The vertebra/rib-aorta distance (mm)	2.3 ± 1.7	2.5 ± 2.0	2.2 ± 1.9	2.7 ± 2.3
Kyphotic angle (°)	80.7 ± 7.1	115.4 ± 13.0^a^	119.6 ± 4.8^b^	129.5 ± 8.1^c^
SVA (mm)	6.8 ± 14.0	5.5 ± 23.2	2.9 ± 22.8	1.5 ± 19.3
ODI	31.3 ± 7.7	30.5 ± 7.4	27.9 ± 8.1	34.8 ± 7.8

*SVA* Sagittal Vertical Axis, *ODI* Oswestry Disability Index

^a^ Indicates a significant difference between Grade2 and Grade1 subgroups

^b^ Indicates a significant difference between Grade3 and Grade1 subgroups

^c^ Indicates a significant difference between Grade4 and Grade1 subgroups

## Discussion

Tuberculosis of the spine is still an international concern, not only in developing countries but is also increasing in developed countries [1]. Progressive bony destruction, a characteristic phenomenon of spinal TB, will collapse the vertebrae leading to severe kyphosis, especially in patients with multiple levels infected. Even with successful medical treatment, progressive severe kyphosis may still develop [11]. For extreme cases, the correction of Pott’s deformity is only feasible with three-column osteotomies [2].

No matter what kind of osteotomies [12–14], these procedures generally improve kyphotic deformity by shortening the posterior column and lengthening the anterior column. Ji et al [12] found that aortic length increased by 2.2 cm averagely after closing-opening wedge osteotomy and the amount of aortic lengthening correlated positively with the degree of kyphosis correction, indicating that the aorta was more vulnerable to get injured in correction of more severe kyphotic deformities. In addition, the procedure of PCR includes a formal costotransversectomy and exposure of the lateral wall of the vertebral body, which could subject the aorta to impingement, especially in patients with grade 3 and 4 aorta (Fig. 2). Although the vascular complication is rare, the consequence may be quite devastating [13–16].

Several authors have studied the position of the aorta relative to the spine in scoliosis. The results of these studies implied that the aorta tends to be located more laterally and posteriorly around the apex, and thus increased injury risk when placing screws in the concave side of scoliosis [9]. Given the pathological nature of Pott’s deformity, the majority of spinal tuberculosis begins in the anterior vertebral body, and the bony involvement in spinal tuberculosis gradually destroys the anterior vertebral bodies, leading to kyphosis. And surgical management is reserved for patients suffering from neurological deficits or severe kyphosis [1]. Thus, in this study, we focused on the position of the aorta relative to the spine in kyphosis secondary to spinal tuberculosis.

Our findings have important implications for osteotomy procedure for Pott’s deformity. Surgeons need to know that the relative position of aorta to the spine may be different in patients with post-tuberculous kyphosis from normal spine. The aorta generally moved to the left side laterally and posteriorly as it descended from T4 and changed its course at T7 and moved medially and anteriorly in normal spine (Figs. 2, 3 and 4) [9, 10, 17], while in Pott’s deformity with only thoracic regions involved, the aorta moved laterally and posteriorly and changed its course at apex level, forming an “Ω” shaped aorta. In this subgroup, the majority of patients presented with grade 2 and 3 aorta, and some with grade 4 aorta, which was absent in group NC. When the osteotomy procedure is performed in patients with grade 4 aorta, the aorta may have a higher risk of injury from the start of the exposure of the rib. In case of the patients with grade 3 aorta, gentle manipulation during the resection of rib heads and the pedicles should be stressed to prevent aortic injury or PSO may be preferable to achieve correction with less risk of the aortic injury. And surgeons should be cautions when removing the lateral aspect of the vertebral body in patients with grade 2 aorta and the anterior part of the vertebral body in patients with grade 1 aorta.

**Fig. 10:**
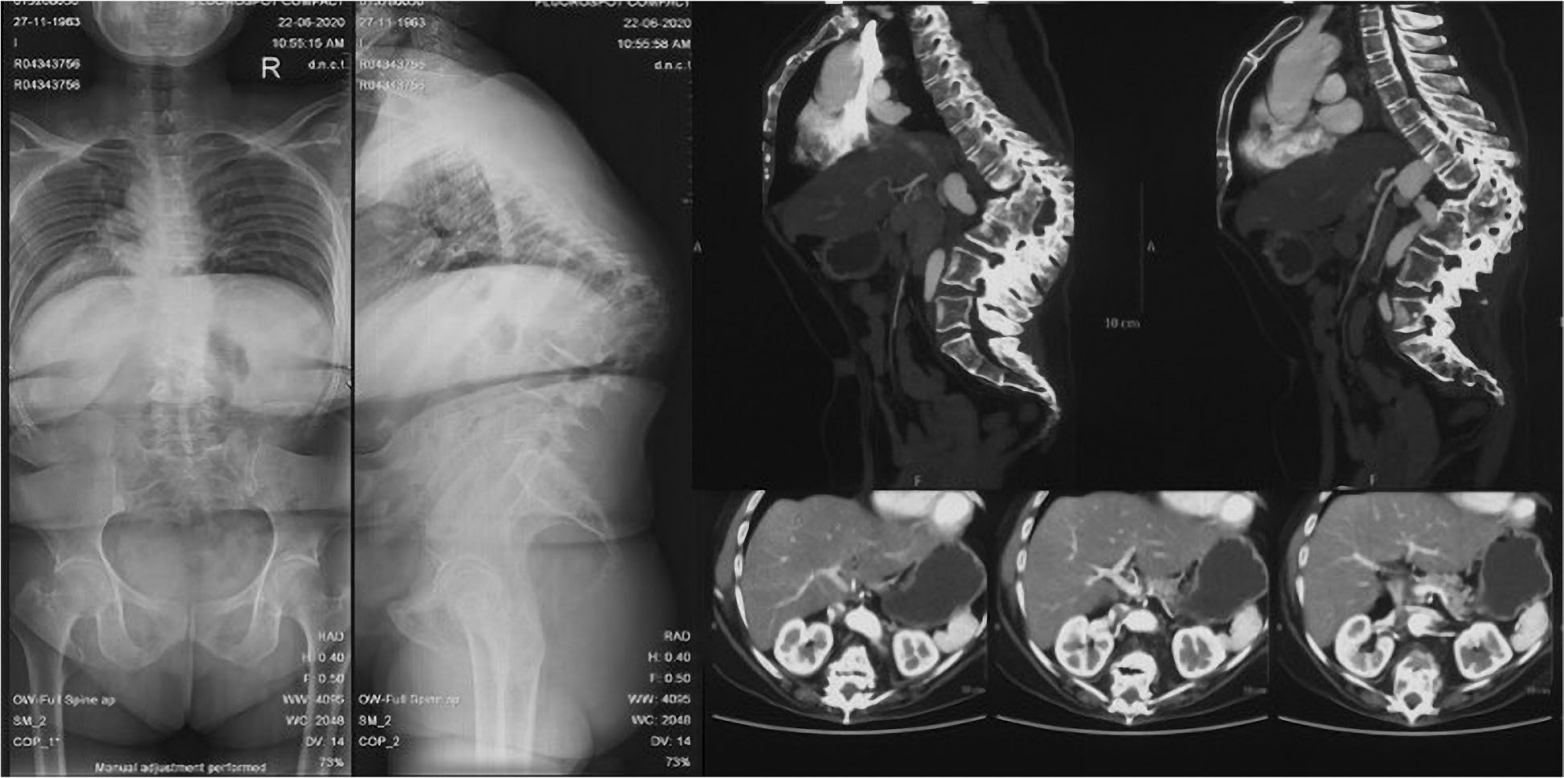
A 57 years old patient presented with numbness of lower limbs. The kyphotic angle of T8-L3 was 96° and SVA was measured 5.0 cm. CT scans showed that the aorta shifted anteromedially to the vertebrae body in at apex vertebral level

Liang et al. [18] analyzed patients with Pott’s thoracolumbar angular kyphosis and found that the aorta is relatively farther away from the fused vertebral body compared with the normal subjects, which is similar with our results of the patients with an “M” shaped aorta (Figs. 5, 10). We assumed that the hiatus of the diaphragm hinders the over-posteriorly moving of the aorta, and provided a larger safe zone for osteotomy procedure in lumbar region. Meanwhile, the aorta was located in proximity to the left aspect of the vertebral body at levels above and below the osteotomy levels, increasing the risk of the aortic injury during left-sided pedicle screw insertion. To avoid aortic injury from osteotomy or pedicle screws inserting, the preoperative CTA scans are recommended, in which the degree of the aorta shifting can be evaluated for the selection of the appropriate osteotomy procedure.

Three potential limitations of this study should be noted. First, the relatively small number of patients were enrolled. Studies of more patients are needed to precisely describe the distribution of different kinds of relative positions of aorta to the vertebrae in normal and post-tuberculous spine. In addition, we excluded patients with scoliosis curve > 10 degrees. Future studies also need to clarify the variation of the position of the aorta relative to a kyphoscoliotic spine secondary to spinal tuberculosis Second, this cross-sectional study cannot address whether the position of the aorta change with aging or kyphotic deformity progression. Moreover, the CTA scans were obtained with the patients in the supine position, while osteotomy were performed with the patients in the prone position. However, no significant change of the relative positions between the aorta and the vertebrae after the patient turned to a prone position in ankylosing spondylitis patients with thoracolumbar kyphosis was reported [19, 20]. Further studies of patients with different positions are needed to address this issue.

## Conclusions

In summary, the results of our study, for the first time, evaluated the relative positions of the aorta to the vertebrae in different kinds of patients with Pott’s deformity (thoracic, thoracolumbar/lumbar kyphosis) and had important implications for osteotomy procedure.

## Data Availability

The datasets used and/or analysed during the current study are available from the corresponding author on reasonable request (dr_zhaojq@163.com).

## Abbreviations

PSO: Pedicle subtraction osteotomy
PVCR: Posterior vertebral column resection
VCD: Vertebral column decancellation
CTA: Computed tomographic angiography
LtP-Ao: Left pedicle-aorta
